# Efficient Resource Allocation for Backhaul-Aware Unmanned Air Vehicles-to-Everything (U2X)

**DOI:** 10.3390/s20102994

**Published:** 2020-05-25

**Authors:** Takshi Gupta, Fabio Arena, Ilsun You

**Affiliations:** 1 Department of Information Security Engineering, Soonchunhyang University, Asan 31538, Korea; takshi_gupta2012@hotmail.com; 2 Faculty of Engineering and Architecture, Kore University of Enna, 94100 Enna, Italy; fabio.arena@unikore.it

**Keywords:** resource allocation, UAVs, Vehicle to Everything (V2X), U2X, load balancing

## Abstract

Unmanned aerial vehicles (UAVs) allow better coverage, enhanced connectivity, and elongated lifetime when used in telecommunications. However, these features are predominately affected by the policies used for sharing resources amongst the involved nodes. Moreover, the architecture and deployment strategies also have a considerable impact on their functionality. Recently, many researchers have suggested using layer-based UAV deployment, which allows better communications between the entities. Regardless of these solutions, there are a limited number of studies which focus on connecting layered-UAVs to everything (U2X). In particular, none of them have actually addressed the aspect of resource allocation. This paper considers the issue of resource allocation and helps decide the optimal number of transfers amongst the UAVs, which can conserve the maximum amount of energy while increasing the overall probability of resource allocation. The proposed approach relies on mutual-agreement based reward theory, which considers Minkowski distance as a decisive metric and helps attain efficient resource allocation for backhaul-aware U2X. The effectiveness of the proposed solution is demonstrated using Monte-Carlo simulations.

## 1. Introduction

Unmanned aerial vehicles (UAVs) are considered to be powerful and versatile in nature, which supports various opportunities related to navigation, surveillance, emergency response, disaster management, health care, urban planning, agriculture, and telecommunications [1,2,3,4,5,6]. To improve, enhance, and optimize industrial processes, UAVs are widely applied for localization and tracking of robotic devices [7,8]. UAVs are capable of capturing information easily by enabling the rapid and seamless data collection that can be regulated to form an automated UAV system.

UAV networking is one of those applications which have enhanced the utility of these vehicles. However, their fully functional usage is limited to certain criteria and network constraints, which include coverage abilities, mobility laws, lifetime, connectivity, hazard tracking, and resource allocation [9,10,11,12]. With a single UAV’s limited monitoring range, several UAVs are required to cover a full cellular setup. This requires solutions to optimization problems, as there is no fixed standard for optimal coverage and network conditions are subject to change with respect to UAVs’ operational states. Mobility is another factor affecting the coverage, which is eminent in defining the lifetime of the networks using UAVs as their core entities [13,14,15]. In addition to these, resource allocation, when UAVs are used as serving gateways, becomes tedious and requires effective policies to regulate the load [16,17]. The use of distributed resource allocation along with energy-efficient schemes can well support such dynamic networks in an optimized manner [18,19,20].

Using UAVs in layers is another approach prominently studied by several researchers, such as Sekander et al. [21], Sharma et al. [22], and Almeida et al. [23]. The authors have shown the abilities of multi-layered UAV architecture in facilitating network load with better efficiency. However, there are limited solutions which dominantly consider UAV to everything (U2X) as a core application scenario. Additionally, with a number of layers of UAVs involved in handling every other entity in the network, the issue of resource allocation becomes very crucial and an effective strategy is required to regulate such networks [24,25].

This article focuses on the issue of resource allocation in multi-layered U2X, and presents evaluations on the number of offloads/transfers which are required to handle the entire area load without any reconfigurations or additional UAVs. Mutual agreement reward theory is used for effectively resolving the optimization constraints. Furthermore, this article considers the backhaul formation between one of the layers of UAVs and the core, which helps to make a decision on reshuffling the UAVs. Such a situation allows for better conservation of energy and a lesser demand for additional support from the network.

### Our Contributions

Based on the problem of resource allocation for backhaul-aware multi-layered U2X, the contributions of this article are summarized below:

- A backhaul-aware U2X scenario with the support of multi-layer UAVs is presented along with a resource allocation problem with the constraints on the number of transfers.
- Mutual agreement reward theory is applied to understand the problem of resource allocation in U2X.
- The entire problem is resolved using coordinated resource allocation, which is accounted for using a reward-jump mechanism.
- Hazard tracking and ownership procedures are used to decide the control over the network and reshuffling amongst layers.
- Monte-Carlo simulations are used to evaluate the proposed approach by generating scenarios with various numbers of failures for different numbers of UAVs.

The remaining parts of the article are organized as follows: Section 2 covers the related works, especially focusing on resource allocation in U2X. Section 3 discusses the system modeling and the proposed solution. Performance evaluations are presented in Section 4, and Section 5 concludes the article.

## 2. Related Works

Several articles have addressed the issue of resource allocation in general UAV networks. Yang et al. [26] focused on the energy-efficient resource allocation in mobile edge networks while considering the power minimization problem for UAV-enabled networks. Their approach is based on the fuzzy c-means clustering-based iterative algorithm. Lemaire et al. [27] focused on the distributed task allocation in UAVs. The authors presented a dynamic task allocation framework based on the incremental task allocation algorithm. The authors pointed out various open issues, such as the robustness against temporally failures and limited communication bandwidth. Furthermore, Li and Han [24] focused on the optimal resource allocation in UAVs for packet delay minimization. The authors used a Voronoi diagram for the system model, and associated properties were used to attain the mean packet data rate. Furthermore, the gradient descent method with the bisection method was used for the minimization of power allocation and optimization of spectra. The optimal node placement problem was studied by Fan et al. [28]. The authors worked on a UAV-relaying network, and a non-convex optimization problem was formulated to increase the system throughput along with resource allocation.

Nguyen et al. [29] worked on the resource allocation problem in D2D communication. The authors considered the maximization of energy efficiency and developed a real-time resource allocation algorithm. Kawamoto et al. [30] emphasized the future aspects of UAVs with the resource allocations and proposed a scheme for a three-UAV network based on the CSMA/CA. The authors used time division-based window of time to avoid conflicts. The authors outlined various problems associated with the real implementation of resource allocation issues in the UAV-aided networks. Hua et al. [31] worked on the energy efficient optimization by considering effective resource allocation, user partitioning, and UAV trajectory. The optimization problem was formulated in the non-convex method and solved through two sub-problems while applying the block coordinates descent method. Sharma et al. [11] focused on the secure resource allocation in aerial-terrestrial networks. The authors presented a novel solution, cog chain with feed and fetch modes, for the content sharing among nodes and the server by using a cog-chain communication protocol. Du et al. [32] emphasized the energy-efficient resource allocation in UAV-based edge systems. The authors considered the hovering time, scheduling, and resource allocation factors for the energy optimization in the network.

UAV backhauling into the core network requires an effective wireless channel for connectivity. The backhaul can be supported by the free space optical (FSO) or millimeter wave (mmWave) connection with a ground station. Galkin et al. [33] used a stochastic geometry to model a UAV network and provided wireless service within coverage areas while ensuring that the UAVs were able to establish a backhaul signal. Fouda et al. [34] focused on the in-band integrated access and backhaul to the UAV-enabled 5G communication. The authors gave an optimization framework to find optimal precoder design for wireless backhaul links, user-base station association, UAV 3D hovering locations, and power allocation for forwarding link transmissions. Sharma et al. [35] focused on the security management for backhaul-aware cellular V2X. The authors discussed the management and security aspects of the backhaul between the terminals and the hub. The authors gave a security framework based on the long and short-range authentications. Furthermore, Galkin et al. [36] focused on the backhaul for low altitude UAVs. The authors considered GS density and antenna characteristics when building density parameters to model the solution for the backhaul in the UAVs. Gapeyenko et al. [37] considered the UAV-based backhaul for the 5G mmWave cellular networks. The authors presented a framework which includes 3GPP’s multipath channel model, and the heterogeneous mobility of UAVs and humans. Choudhary et al. [38] explored security issues for the backhaul approach based on 5G mmWave cellular networks, which need to be considered for UAV-based 5G backhaul.

UAV-to-X Communications have increased rapidly and have been effectively applied in military, public, and civil applications. Zhang et al. [39] gave a cooperative UAV sense-and-send protocol to support UAV-to-X communications. To enhance the uplink sum-rate, the authors formulated a sub-channel allocation and UAV speed optimization problem and gave an iterative sub-channel allocation and speed optimization algorithm (ISASOA) as a solution. In their earlier works, Zhang et al. [40] focused on the maximization of the uplink sum-rate through resource allocation and trajectory design. The authors gave an iterative subcarrier allocation and trajectory design algorithm (ISATCA) for solving the joint subcarrier allocation and trajectory design problem. Additionally, a comparative study, summarizing these existing solutions, is presented in Table 1.

## 3. Proposed Approach

This section presents a detailed system model and formulates the actual problem to be solved as a part of energy-efficient resource allocation. A coordinated proposal is used for solving the resource allocation problem while considering the scenarios with increasing as well as decreasing failure rates. This section also explains how the traffic is handled and how the system adjusts itself to accommodate failures while keeping intact the constraints defined in the system.

### 3.1. System Model and Problem Formulation

**Figure 1:**
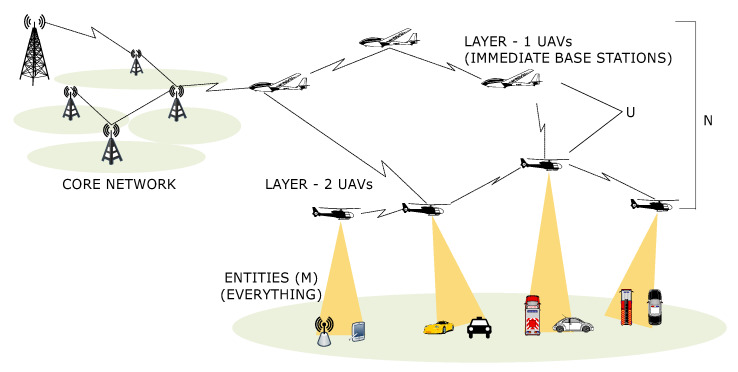
An exemplary illustration of a multi-layer U2X scenario for resource allocation.

The network is considered to be operating in U2X mode, where each UAV is capable of communicating with every other device active in the network. Additionally, the network uses multiple tiers of UAVs to formulate a resource allocation problem that primarily considers energy-optimization as a backbone solution for communication between the entities, as shown in Figure 1. For this, let N be the set of *N* tiers into which the UAV network is divided. These UAVs communicate with a set M of everything-containing *M* devices, which includes user equipment or base stations. UAVs in each layer are represented by a set $U_{<N>}$, where *U* refers to their numbers. The entire system is presented as an inverse problem for offloading, which decides the number of transfers of load (δ) under which the U2X network can perform effectively at minimum consumption of energy (E), low latency (L), low scheduling overheads (S), lower per-UAV load (γ), and maximum allocation of resources (R). This problem can be written as:

(1) $$P:\min\left(\delta \right)\mathrm{and}\max\left(R\right),$$

subject to

(2) $$\begin{array}{cc} C1: & \min\left(E\right),\forall U\in U,\forall N\in N,\forall M\in M,\\ C2: & \min\left(L\right),\forall U\in U,\forall N\in N,\forall M\in M,\\ C3: & \min\left(S\right),\forall U\in U,\forall N\in N,\forall M\in M,\\ C4: & \min\left(\gamma \right),\forall U\in U,\forall N\in N,\forall M\in M. \end{array}$$

To formalize the above issue, let $C_{X}$ be the number of devices a lower tier UAV can handle and $C_{U}$ be the number of UAVs an upper layer UAV can accommodate to compensate the connectivity across the network. Note that the number of layers has a sufficient impact on the system and it affects additional aspects, such as received power, channel loss, and interference-mitigation, to be resolved explicitly, which are beyond the scope of this article. Although generic, this article predominantly focuses on using two tiers of UAVs. Based on this, the entire network must comply with $C_{<P>,X}\leq C_{X}$ and $C_{<P>,U}\leq C_{U}$ at any instance, where, subscript <P> refers to the present demand of connections. The proposed model computes E by considering the maneuvering energy of UAVs ($E_{M}$), link formation energy ($E_{N}$), transmission energy ($E_{T}$), and scheduling energy ($E_{S}$), such that

(3) $$E=\sum_{i=1}^{U}{\left(E_{M}\right)}_{i}+\sum_{j=1}^{|C_{T}|}{\left(E_{N}\right)}_{j}+\sum_{k=1}^{|C_{A}|}{\left(E_{T}\right)}_{k}+\sum_{q=1}^{U^{\prime }}{\left(E_{S}\right)}_{q}+E_{\epsilon },$$

where $C_{A}$ and $C_{T}$ are the number of active links and total links between the entities, respectively, and $U^{\prime }$ is the UAVs involved in the decision making process for handling everything. Additionally, the replacement energy ($E_{\epsilon }$) is included to facilitate the failure rate in the network. Now, L includes several metrics [41] like transmission latency ($L_{T}$), decision latency ($L_{D}$), and replacement latency ($L_{R}$), such that

(4) $$L=\left(\frac{1}{C_{\mathrm{OA}}}\sum_{i=1}^{|C_{A}|}{\left(L_{T}\right)}_{i}\right)+L_{D}+L_{R},$$

where $C_{\mathrm{OA}}\;(\leq |C_{A}\left|\right)$ represents the number of common active links, $L_{D}$ and $L_{R}$ are defined for maximum value amongst all entities. Here also, $L_{R}$ is calculated based on the failure rate of entities, and $L_{T}$ is calculated as the sum of the queuing delay and propagation delay, and it may vary for UAV to UAV links and UAV to X links. The per-UAV load can be expressed using a load function $F_{U2X}$, such that

(5) $$F_{U2X}=\frac{1}{U_{H}^{\left(C\right)}}\int_{\left(x\in A)\forall (U\in U\right)}\frac{O_{R}}{W\log(1+\mathrm{SINR})}\mathrm{dx},$$

where SINR is the signal to interference plus noise ratio, $U_{H}^{\left(C\right)}$ is the handling capacity of each UAV irrespective of its tier, $O_{R}$ is the offered transmission rate, *A* is the total deployment area, *x* denotes the present location of the UAV under evaluation, and *W* is the bandwidth.

To model δ and R, a mutual agreement reward theory inspired by “contract theory” [42] is formulated following the network state and considers the failure-policies. The failure-policies are the ones which manage the increasing or decreasing rate of non-available entities in the network. In the model, a hazardous constant (ψ) is derived based on failure-policies, which maintains the flow of mutual agreement across the UAVs for coordinated resource allocation. As $U^{\prime }$ is the number of decision taking UAVs, it is expected that a similar number of UAVs are required to continue the operations in the network. Because of that, based on neoclassical model [42], the utility of the network can be given by

(6) $$Y=\sum_{i=1}^{U^{\prime }}{\left(F_{U2X}\right)}_{i}{\left(H\right)}_{i},$$

where H defines the network cost to handle $F_{U2X}$, and it is given as a time function of energy, minimum rate, and duration, which can be written as $H=T(E,R_{\min},\tau )\pm \psi$. Here, $R_{\min}$ is the minimum transmission rate and τ is the operational duration of the network. To simplify, this function can be modeled using logistic distribution [43] as it provides a better understanding of the situations where unpredicted failures are involved. By definition [44],

(7) $$H=\frac{e^{\eta }}{\tau_{\psi }{\left(1+e^{\eta }\right)}^{2}},$$

where

(8) $$\eta =\frac{\tau -\tau_{E}}{\tau_{\psi }},\tau_{\psi }>0,\tau \neq 0.$$

Here, $\tau_{E}\left(\leq \tau \right)$ refers to the duration a network can sustain the condition $E\leq E_{A}$, which is the available energy along with the maintenance of $R_{\min}$, and a situation $\tau_{E}=\tau$ requires no offloading (ideal case). $\tau_{\psi }$ is the difference between the actual time and the instance referring to change in failure-policies; i.e., time at which ψ≠0. This entire model can be logistically controlled using Chebyshev distance [45], which helps to define R, such that

(9) $$R=\lim_{\tau \to \infty }{\left(\sum_{i=1}^{\omega }{\left|M_{i}-M_{i}^{\prime }\right|}^{\tau }\right)}^{\frac{1}{\tau }},$$

where *M* and $M^{\prime }$ are the total and unhandled entities during the *i*th instance. ω defines the step interval for the operational time τ. Equation (9) can also be modeled around failures, where ψ (ψ≠0) can be specifically accounted as $⎡{\left(\frac{U-U^{\prime }}{U}\right)}^{-1}⎤$, such that R changes to a Minkowski distance [46] with a value ${\left(\sum_{i=1}^{\omega }{\left|M_{i}-M_{i}^{\prime }\right|}^{\psi }\right)}^{\frac{1}{\psi }}$. Using their definitions [45,46], the problem statement can be further simplified as follows:

(10) $$P1:\max\left(|M_{i}-M_{i}^{\prime }|\right),\forall \omega ,$$

subject to

(11) $$\begin{array}{cc} C1: & \min\left(\delta \right),s.t.\left(2\right),\\ C2: & \min\left(Y\right),\forall U,\\ C3: & \tau -\tau_{E}\geq 1,\psi \geq 1. \end{array}$$

The optimization variable in (10) follows (9), which can be maximized by reducing the number of unhandled entities in a given instance.

### 3.2. Coordinated Resource Allocation

The proposed approach supports efficient resource allocation while considering backhaul-enabled multiple UAVs connected to every entity in the network. The backhaul facilitates continuous support for UAVs to take appropriate decision on sharing the load and it helps to resolve the constraints associated with the optimization problem expressed in the earlier subsection. Following points should be considered while deploying the proposed coordinated resource allocation solution and attaining an efficient resource allocation scheme:

- The proposed approach aims at maximizing the resources handled by UAVs while decreasing the exchange or limiting the shifting users across the UAVs, except for the scenarios wherein UAVs fail in batches. Irrespective of such failures, the proposed approach controls the network and offloads the traffic with limiting iterations.
- For multiple tiers scenarios, the UAVs which support the backhaul links are the ones which make a decision on the load sharing capabilities of fronthaul UAVs by considering U, M, and N, whereas for scenarios with single tier UAVs, the decision on load sharing is only taken based on the mutual agreement reward theory only between U and M. It is to be considered that the former offers better evaluation in case of failures, while the latter is effective in low-overheads with less resistance to failures.

**Figure 2:**
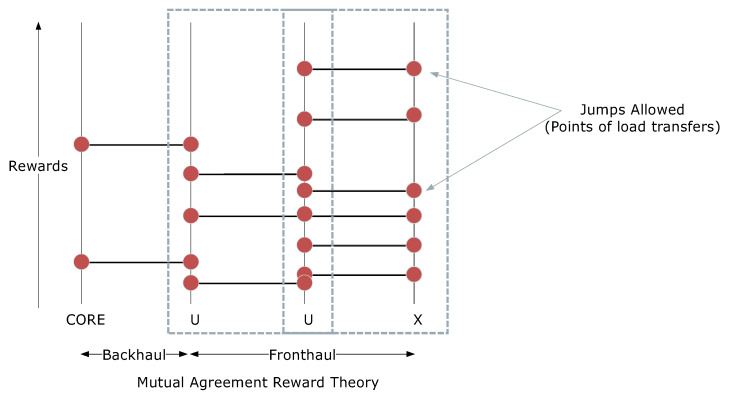
An exemplary illustration of reward-jump system used for mutual agreement between the entities to ensure smooth and low-overhead offloading.

To facilitate the entire process, the reward-jump associations are formulated based on the earlier expressed mutual-agreement reward theory. According to this, each entity in the network accumulates certain reward values, which are helpful in shifting load across each other. This shifting is referred to as jumping, hence the name reward-jump mechanism. An illustrative diagram for this is shown in Figure 2. The rewards are given whenever the associated entities resolve the earlier formed optimization problems and obey the system constraints. It is to be taken into consideration that reward gathering is considered one task, while the decision on offloading is a separate task; however, the decision on offloading is only made when an entity has a sufficient number of reward values. The details on these formulations for each of the connections in the given network are provided below:

- Let $J_{R}$ be an incremental reward function with an initial value at 0, which increases as the number of times the network obeys the set constraints. This increment is the actual value assigned to $J_{R}$, and it may vary for different scenarios. In certain cases, it may follow an increment or decrement value depending on the failure rate of the network. Additionally, the reward function may vary for the types of entities and their connectivity as shown in Figure 2.
- Once $J_{R}$ is defined, a threshold is set for each of the categories and the network is evaluated against it to allow a decision on load balancing/resource allocation/offloading.
- The value of $J_{R}$ is also used to take a critical decision of the entity, which will be dominating the decision on resource allocation.

All the above aspects help to provide coordinated resource allocation that involves coordination between the core, layers of UAVs, and the end-user entities.

### 3.3. Backhaul Coordination

The failures are caused in the network because of improper management of resources. In particular, when the network entities do not consider the backhaul, the decision for offloading may look fine from fronthaul’s perspective, but it may induce extra overheads on the companion nodes. Thus, it is desired to consider resource allocation as a joint problem throughout the network. To resolve this, we employ the use of intermediate UAVs help to perform inter-cell coordination. This allows for better decision making to edge UAVs, which primarily serves the end-user entities. Furthermore, with the use of intermediate UAV layers, the single point of failure and excessive loads on a common node can be avoided, as in certain cases, the UAVs can even exchange their positions between the layers to facilitate better connectivity along with the maximum conservation of energy.

Furthermore, in the proposed approach, the backhaul coordination is done only by using UAVs, which have better lifetime and maximum coverage. Here, coverage refers to the awareness of a UAV with respect to the other components and their operational metrics. Such a mechanism helps to generate accurate reward functions. This can be understood with the following example: consider a situation wherein the number of end-users increases beyond the capacity of near-user UAVs. In such a situation, the network optimization criteria may fail and there is no longer facilitation of constraints. Thus, the choices may involve either increasing the capacity of UAVs or deploying more UAVs. The second solution is widely preferred in the majority of the existing solutions; however, in the proposed approach, a similar strategy is used, but the UAVs are selected from the ones which support the backhaul and have better reward function values. Overall, the network tries to maintain a balance of reward function across the layers so as to provide better load-sharing whenever such a situation arises.

### 3.4. U2X Coordination

Once the backhaul is set to facilitate requests for moving UAVs between layers, the system is set to perform resource allocation while lowering the overall consumption of energy. Moreover, E function in coordination with δ to take appropriate decision on when to shift the load and where to shift the load.

To allow this functionality, the resource allocation module is installed on all the UAVs where a special flag is used to check if the UAV operates for fronthaul or backhaul operation. As a part of backhaul, the system maintains an account of the overall consumption of the entire layer of UAVs. This helps to check whether the network can continue to operate or additional resources are required. Once this condition is met, the network traces individual UAV and checks for requests handled by them while offering reward points after certain time intervals. Once the UAVs have sufficient reward points, it advertises itself for offloading. Finally, UAVs with fears of reaching thresholds check for the advertised UAVs and offload traffic in such a way that minimum energy is consumed and maximum reward points are saved.

Each time an exchange is done, the reward-jump value of the involved entities is decremented as per the reward function, and the system continues to settle for the minimum operational requirement. In no instance will the value of the reward function refer to the situation of zero-tolerance, as new UAVs have to be accumulated or load has to be increased on certain UAVs. In such a situation, the overall reward value of the system may decrease, but this can prevent the loss of connectivity and avoid any isolation. The decrease in the values leads to penalties and its far lower value can result in major shutdowns. Thus, it is desirable to transfer load appropriately for which the thresholds for the reward function must be selected effectively.

### 3.5. Hazard Tracking and Ownership

It is required to quantify the network performance, which can be done using hazard tracking and penalties. These help ascertain that the proposed approach always makes an accurate decision for resource allocation and the correct entity is in-charge of the decisions made. To further understand the hazard tracking and penalties, two conditions, as shown below, are derived. These conditions help to understand how ownership of decision making is decided and the optimal points at which the given network can perform without inducing additional failures.

Condition-1: This condition deals with the optimal ownership with hazards (failures) for the UAVs across which the load is to be divided. This operates alongside reward-jump function, and according to this, the entities with $\min\left(H\right)$ offer better operability in the network and are used for making a decision on the resource allocation. The entities which satisfy this condition are further evaluated to check if these can help with sustaining the connection. This can be further defined by changing η in (7) to the actual state of operations (by using ψ) and performing predictability based on the failure rate in the network. According to this, the entities with $\min\left(|H-H_{p}|\right)$ can make the final decision for allocating the resource and shifting the load in decreasing order of the resultant. Here, $H_{p}$ can be evaluated as $\frac{e^{\alpha }}{\tau_{\psi }{\left(1+e^{\alpha }\right)}^{2}}$, where α is the predicted constant for UAVs such that

(12) $$\alpha =\int_{\tau }\frac{U-\min{\left(U\right)}_{t}}{U-\lambda e^{-\lambda t}}\mathrm{dt},$$

where λ is the hazard rate function for the number of UAVs failing during the given interval, $\lambda e^{-\lambda t}$ is the failure rate of the UAVs, and $\min{\left(U\right)}_{t}$ is the minimum number of required UAVs during the given instance. Equation (12) on evaluating in a definite interval gives $\left(U-\min{\left(U\right)}_{\tau }\right)\left(\frac{\ln\left(|\lambda e^{-\lambda \tau }-U|\right)-\ln\left(|\lambda -U|\right)}{\lambda U}+\frac{\tau }{U}\right)$ as an output, which is used to decide the dominance of a UAV in taking the ownership of the network.

**Figure 3:**
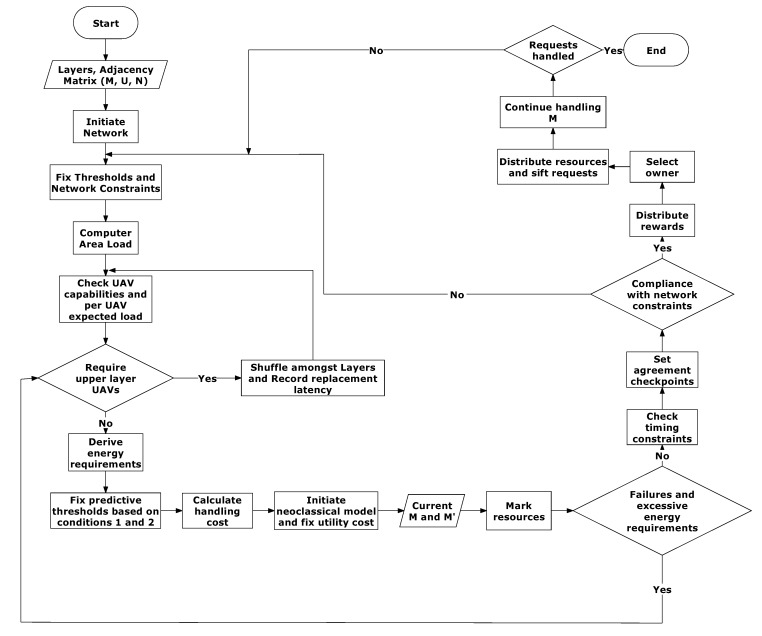
Flow chart representing all of the procedures and work flow for energy efficient resource allocation in U2X.

Condition-2: This condition deals with the maximum number of transfers for allocating the maximum resources in contrast with the optimal ownership with hazards. For this, limits are set for R and Y, which are represented by $R^{\prime }$ and $Y^{\prime }$, respectively. These values can either be system thresholds or average values attained from the previously observant states. Based on these conditions, δ has its values in the range between the number of times the ownership is changed and the number of times the utility cost becomes more than the permissible values alongside decrements in the required value of R. At any instance in the network, successful operations are marked at $R>R^{\prime }$ and $Y\leq Y^{\prime }$. Since change in ownership does not always require a shift in load, the value of δ can be predicted to be greater than equal to the number of times the ownership is changed and it is predicted to not go beyond the number of times the conditions ($R>R^{\prime }$ and $Y\leq Y^{\prime }$) are unsatisfied. Thus, at any instance, if δ breaks the upper limits, the network cannot function at its given specifications and reconfigurations (including additional use of UAVs) may be required. Such a condition is extreme and may cause additional overheads. However, a continuous reshuffling (after certain timestamps) can be performed to move UAVs across the layers, as this allows maintenance of the overall utility cost which keeps the number of offloads well under limits. The details of the entire model can be seen in the flow chart given in Figure 3. Based on the system model, the network cost follows a minimum transmission rate, which operates with the complexity of $O(\log\left(R_{\min}\tau \right))$, whereas the deployment complexity of the network is of the order O(UN). Similar to this, the energy optimal conditions follow $O(UC_{T})$.

## 4. Performance Evaluation

The proposed approach is verified by using Monte-Carlo simulations for its efficacy in handling requests and allocating resources while conserving the maximum amount of energy at an optimal number of transfers. The Monte-Carlo simulations were fixed in checking whether more UAVs should be deployed or not. Further, the inclusion of reward functions is done with probabilistic variation in shifting services across the UAVs based on the induced number of failures. These evaluations allow for the understanding the impacts of constraints which affect the mutual agreement reward theory amongst the UAVs and everything. The details of configurations used for evaluations are given in Table 2. The network is operated with each handling UAV having a maximum energy equivalent to 285 MJ.

**Figure 4:**
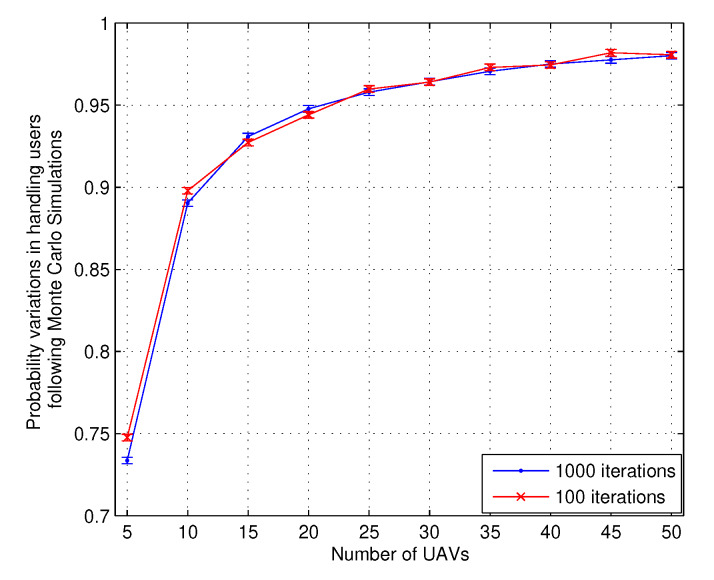
Probability variations for Monte-carlo based simulations vs. Number of UAVs.

The results were achieved with 100–1000 runs. However, the variations based on the number of runs were limited; hence, all the major evaluations are presented for 100 runs. To support this argument, a graph is presented in Figure 4, which shows the variation in handling user requests with an increasing number of UAVs for two different number of runs. Both the scenarios operated with an error of 0.002, and difference in observations is 0.24 %, which is acceptable and it can be stated that the behavior of the approach does not change with the variation in the number of runs. However, the number of runs definitely associates with the clustering of resultant values and producing far better outputs. Such a situation is taken care of in the article and wherever applicable results are presented for the different number of runs.

**Figure 5:**
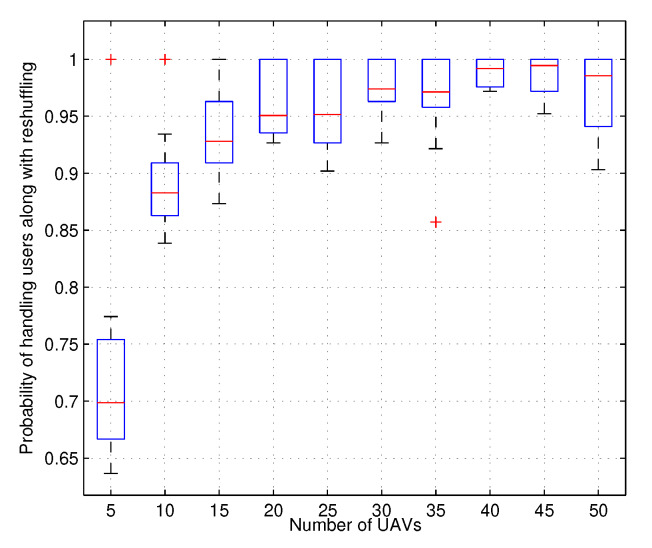
Probability of handling users with reward-based reshuffling vs. number of UAVs.

**Figure 6:**
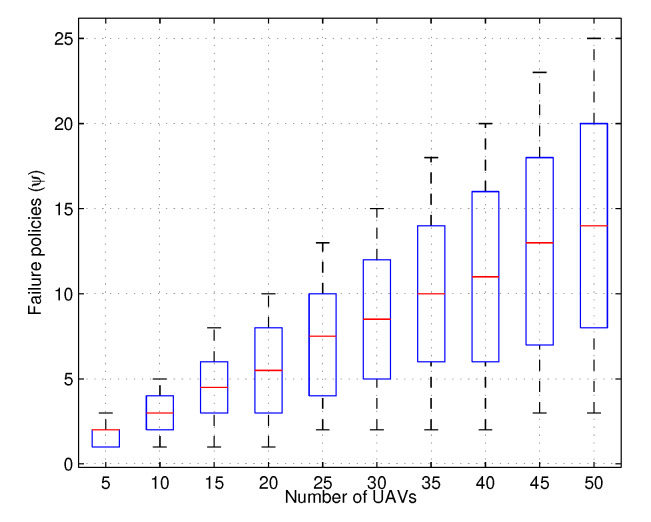
Induced failure policies vs. number of UAVs.

It is to be noted that with the use of reward-based mutual agreement, the optimization problem converges far better and the probability of handling users at the given number of resources increases if the network is allowed to reconfigure. However, such a state is also affected by the number of failures and the current range of active links. Despite that, the proposed approach shows an increasing value in the probability of handling users with an increasing number of failures against the fixed number of UAVs, as shown in Figure 5. The failed policies used to trace this result are shown in Figure 6. Both these graphs help understand the situation under which the Monte-Carlo simulations were applied. Additionally, the shuffling and no-shuffling of UAVs were also considered while tracing the results. It was evidently traced that with the inclusion of shuffling between the layers, the resource allocation increases and the network conserves much of its resources.

**Figure 7:**
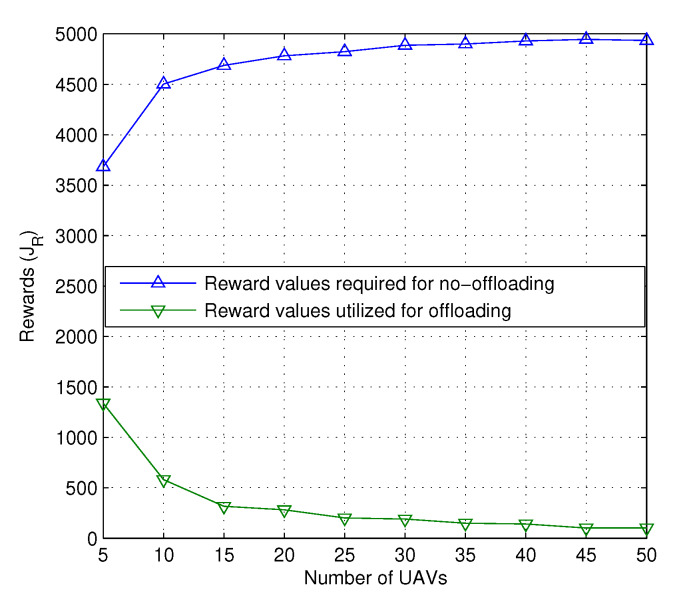
Rewards vs. number of UAVs.

In case the network wants to continue without reshuffling to avoid replacement latency and decision overheads, a certain number of reward values are required. As stated earlier, these reward values are accounted for by the number of times the UAVs in the network obey the constraints of the network. For tracing up to milliseconds at a total run time of 1000 s, the number of rewards required to maintain the state of the network is 92.75% higher for a general scenario than the proposed situation, which performs better resource allocation at lower values of reward function for an increasing number of UAVs, as shown in Figure 7. These reward functions definitely affect the number of transfers required for the continuity of the network. With an increasing number of UAVs, the number of transfers is bound to decrease. However, the number of failures causes a considerable impact on the number of transfers and increases as the number of induced failures increases. This also impacts the latency while requiring additional overheads in sending UAVs to a new location. However, results for replacement latency are not studied at the moment and the major focus is given to understanding whether the network can serve the users with current configurations without requiring additional UAVs or redeployment.

**Figure 8:**
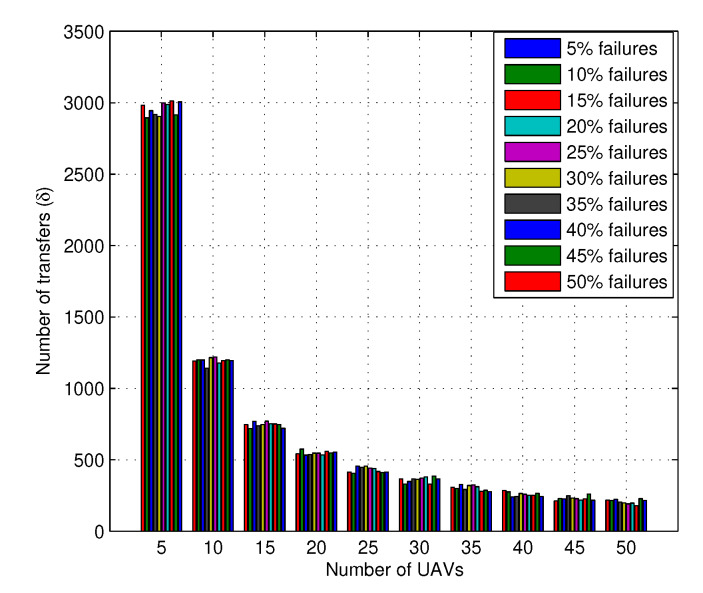
Number of transfers vs. number of UAVs at varying percentages of failures.

**Figure 9:**
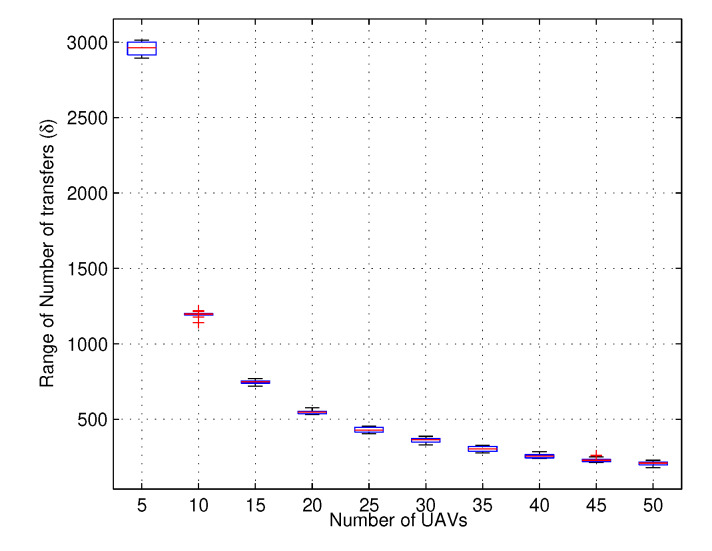
Range of Number of transfers vs. number of UAVs at a fixed number of failures.

The results for the number of transfers with an increasing number of failures for different scenarios of UAVs are shown in Figure 8 and Figure 9. The results suggest that with a variation in the number of failures is from 10% to 50% of total UAVs and the number of transfers is affected by 2.98%, whereas a single scenario with a fixed number of UAVs may require 92.99% more transfers to accommodate requests. Thus, it is to be understood from this that the network with multiple UAVs and layers require efficient configurations, and if the number of failures is allowed go beyond certain values, the network becomes unstable. Moreover, the number of failures U2X can handle without affecting its performance is another issue to resolve, which is left as a part of our future works.

**Figure 10:**
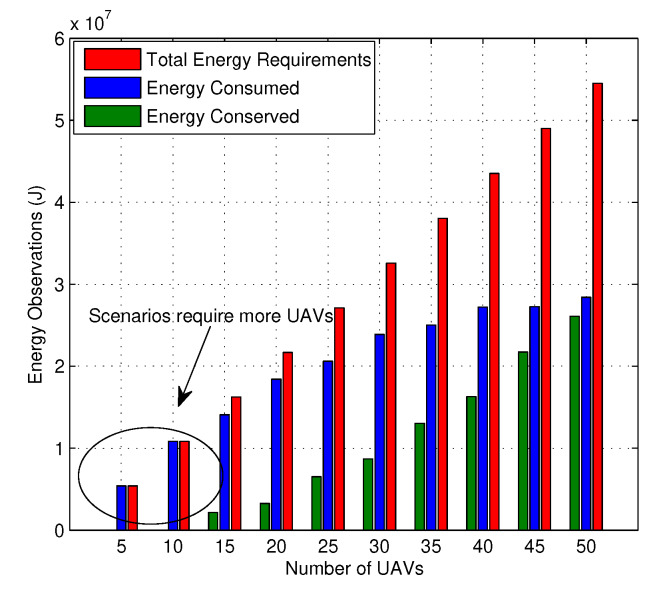
Energy conservation vs. number of UAVs.

The entire network functions towards maximum allocation of resources. But these issues have a huge impact on the energy model of the network, and it is required to understand how well the network utilizes its energy while allocating the resources. As shown in Figure 10, the network operating with 5 and 10 UAVs at a fixed user load has to utilize all of its resources and require additional energy for handling its users. However, with an increasing number of UAVs and more available energy, the proposed solution results in lower consumption and maximum conservation of energy. It also suggests that with an increase in the number of failures, the network can still operate with sufficiently high conservation of energy by controlling the number of transfers required to accommodate all the user requests.

**Figure 11:**
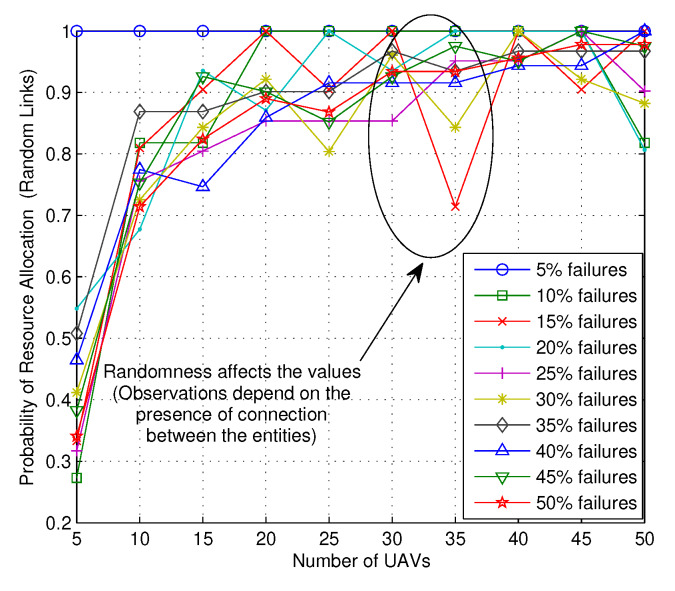
Probability of resource allocation with random link deployment vs. number of UAVs.

**Figure 12:**
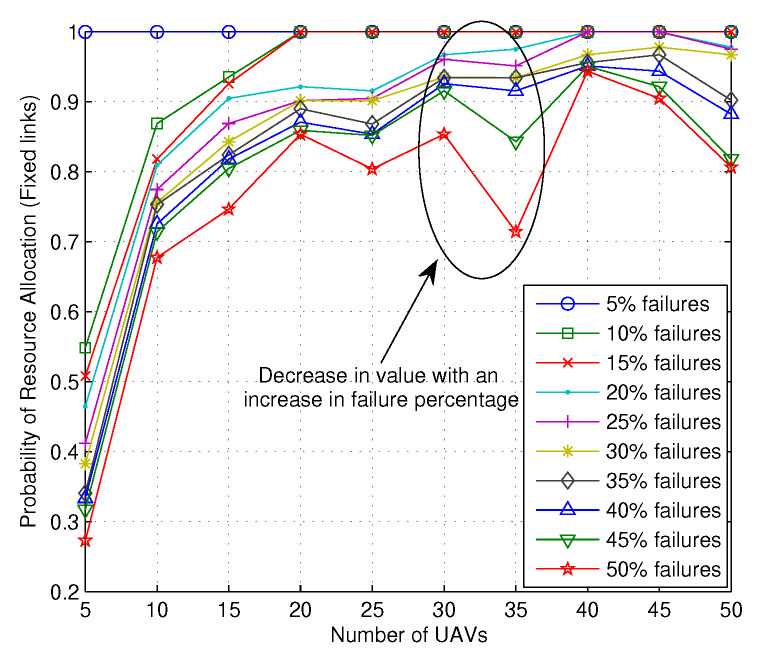
Probability of resource allocation with fixed link deployment vs. number of UAVs.

Finally, to present the efficacy of the proposed solution, results are traced for the probability of resource allocation with random and the fixed numbers of links for an increasing number of failures over the increasing number of UAVs, as shown in Figure 11 and Figure 12, respectively. Irrespective of the scenarios, it can be noticed from both the figures that the number of failures has a major impact on the resource allocation and it decreases if the number of failures is increased for all the networks operating with a different number of UAVs. However, with more UAVs, the network has the flexibility of reshuffling UAVs across its layers and allocates UAVs with better reward values, which increases the overall probability of resource allocation. With extremely low failure rates, the numbers of reward-jumps are minimal; hence, the probability is close to 1 while 50% of network issues lowers the probability of resource allocation to as low as 0.3. Hence, following the argument for Figure 10, network planning and initial configurations play crucial roles in handling user requests and efficiently allocating resources across its entities. Irrespective of these, certain aspects like operational latency, decision-making, and the role of ownership are left to further evaluations and will be discussed in our future reports.

## 5. Conclusions

This paper considered the issue of resource allocation in backhaul-aware U2X. The proposed approach is useful in deciding the optimal number of transfers amongst the UAVs, which can conserve the maximum amount of energy while increasing the overall probability of resource allocation. The proposed approach allocates reward-jump points based on mutual agreement reward theory which helps shuffle UAVs across multiple layers. Such an approach effectively reduces the requirements of additional network support and UAVs and decreases the number of transfers to handle all the user requests. Monte-Carlo simulations were conducted to prove the effectiveness of the proposed approach. Results were traced for the number of rewards required to attain high probability in resource allocation at a lower number of transfers. Furthermore, the results showing impacts on the probability of handling users, resource allocation, and energy consumption with an increasing number of failures over the varying number of UAVs were also presented. The results suggest that the proposed approach can readily decrease the rewards’ requirements by 92.75% compared to a general setup with no-reshuffling and impact the number of transfers by 92.99% by using layered UAVs along with reshuffling in comparison with the fixed number of UAVs without reshuffling.

In the future, this work will be extended to include the study of latency aspects, optimal ownership, and sustainability against the number of failures. 

## Figures and Tables

**Table 1:** Comparative summary of existing works. (R1: resource allocation, R2: backhaul).

Articles	Key Contribution	R1	R2	U2X
[26]	Energy-efficient resource allocation scheme	Yes	No	No
[27]	Distributed tasks allocation scheme	Yes	No	No
[24]	Resources allocation mechanism for minimize packet transmission delay	Yes	No	No
[28]	Node placement and communication resource allocation scheme	Yes	No	No
[29]	Real-time optimal resource allocation	Yes	No	No
[30]	Resource allocation for a three-UAV network	Yes	No	No
[31]	Energy-efficient optimization scheme	Yes	No	No
[11]	Secure resource allocation scheme in ATCNs	Yes	No	No
[32]	Energy efficient resource allocation	Yes	No	No
[33]	Stochastic geometry model of backhaul	No	Yes	No
[34]	In-band integrated access and backhaul management	Yes	Yes	No
[35]	Security management scheme for backhaul-aware C-V2X	No	Yes	No
[36]	Model for wireless backhaul to UAVs	No	Yes	No
[37]	UAV-assisted backhaul scheme for 5G mmWave Cellular Networks	No	Yes	No
[39]	Optimized cellular UAV-to-X communications	Yes	No	Yes
[40]	Resource allocation and trajectory design	Yes	No	Yes

**Table 2:** Simulation parameters.

Parameter	Values	Description
*N*	2	Number of tiers
*M*	100–1000	Number of devices/entities
*U*	10–50	Number of UAVs
$E_{M}$	1.08× $10^{6}$ J	Maneuvering energy of UAVs [47]
$E_{N}$	0.32 J + 0.25 J	Link establishment
$E_{T}$	0.32 J	Transmission energy
$E_{S}$	1 J	Scheduling energy
τ	1000 s	Total operational time
$L_{T}$	20–40 ms	Transmission latency [48]
$L_{D}$	0.5 s	Decision latency
$L_{R}$	0.5 s	Replacement latency
ψ	5–50% of *U*	Hazardous constant
*A*	2500 sq.m	Area under evaluation
*W*	100 MHz	Bandwidth
$O_{R}$	200 Mbps	Offered rate
SINR	100	SINR
$U_{H}^{\left(C\right)}$	200 kbps–200 Mbps	per UAV handling capacity
ω	10	Step interval
